# NECTIN-4-redirected T cell Antigen Coupler T cells bearing CD28 show superior antitumor responses against solid tumors

**DOI:** 10.3389/fimmu.2024.1456443

**Published:** 2024-12-13

**Authors:** Cheng Wei, Xin Huang, Tianlong Xu, Yinan Fang, Fabao Wang, Qiaolin He, Peiyuan Zhang, Qianjin Yu, Ying Zhang, Binjiao Zheng, Yue Gao, Yongping Chen, Qichuan Zhuge, Ai Zhao, Jimin Gao, Jinhong Jiang

**Affiliations:** ^1^ Zhejiang Provincial Key Laboratory of Aging and Neurological Disorder Research, The First Affiliated Hospital of Wenzhou Medical University, Wenzhou, China; ^2^ Key Laboratory of Laboratory Medicine, Ministry of Education, School of Laboratory Medicine and Life Sciences, Wenzhou Medical University, Wenzhou, China; ^3^ Department of Geriatric, Affiliated Hangzhou First People’s Hospital, Zhejiang University School of Medicine, Hangzhou, China; ^4^ Hepatology Diagnosis and Treatment Center, The First Affiliated Hospital of Wenzhou Medical University & Zhejiang Provincial Key Laboratory for Accurate Diagnosis and Treatment of Chronic Liver Diseases, Wenzhou, Zhejiang, China; ^5^ Zhejiang Qixin Biotech, Wenzhou, China; ^6^ Department of Hematology, The Sixth Affiliated Hospital of Wenzhou Medical University, Lishui, Zhejiang, China

**Keywords:** T cell Antigen Coupler (TAC-T), CD28, NECTIN-4, solid tumor, adoptive cell transfer therapy

## Abstract

**Introduction:**

T cell Antigen Coupler (TAC) T cells harness all signaling subunits of endogenous T cell receptor (TCR) to trigger T-cell activation and tumor cell lysis, with minimal release of cytokines. Some of the major obstacles to cellular immunotherapy in solid tumors include inefficient cell infiltration into tumors, lack of prolonged cellular persistence, and therapy-associated toxicity.

**Methods:**

To boost the cytotoxic potential of TAC-T cells against solid tumors, we generated a novel NECTIN-4-targeted TAC-T variant, NECTIN-4 TAC28-T, which integrated the co-stimulatory CD28 cytoplasmic region, and compared the anti-tumor activities between NECTIN-4 TAC-T cells and NECTIN-4 TAC28-T cells in vitro and vivo.

**Results:**

We demonstrated NECTIN-4 TAC28-Tcells could be effectively activated by NECTIN-4 protein-coated magnetic beads (NECTIN-4-beads), and further revealed that the incorporated CD28 co-stimulatory domain enhanced their activation and proliferation capabilities. Notably, NECTIN-4 TAC28-T cells exhibited better anti-tumor effects both in vitro and in vivo than the original NECTIN-4 TAC-T cells.

**Discussion:**

Our data highlighted that NECTIN-4 TAC28-T cells may represent a promising, safe and effective cell therapy for NECTIN-4-overexpressing solid tumors.

## 1 Introduction

In the past few decades, the potency of the adoptive cell therapy (ACT), including tumor-infiltrating lymphocytes (TIL), TCR gene therapy, NK cells, chimeric antigen receptor (CAR)-T or NK cells, etc., in the treatment of cancer has been a major focal point of research (1, 2). CARs activate T cells to lyse target cells in an MHC-independent manner, and CAR-T therapy has achieved revolutionary success in the treatment of hematological malignancies (3, 4). However, mortality during the treatment can occur due to two main complications: cytokine release syndrome (CRS) and immune effector cell-associated neurotoxicity syndrome (ICANS) (5–7). In recent years, a new type of chimeric receptor called T cell antigen coupler (TAC) has been reported. TAC can use endogenous TCR to transduce signals, including three parts: a single-chain variable fragment (scFv) targeting the antigen, a scFv binding to CD3 and a CD4 cytoplasmic domain, which has stronger cytotoxicity, less cytokine secretion, and no tonic signal compared with the second-generation CARs (8, 9).

NECTIN cell adhesion molecule 4 (NECTIN-4) is a type I transmembrane protein consisting of three extracellular immunoglobulin domains, a transmembrane helix and an intracellular domain (10). NECTIN-4 is barely expressed in adult healthy tissues but highly expressed in various tumors (11–13). Padcev (also called enfortumab vedotin), a NECTIN-4-directed antibody and microtubule inhibitor conjugate, has been approved by FDA for the treatment of adult patients with locally advanced or metastatic urothelial cancer who had previously received a PD-1/PD-L1 inhibitor and a platinum-containing chemotherapy (14, 15). We previously reported NECTIN-4 CAR-T cells co-expressing IL-7 and CCL-19 displayed significant anti-tumor activity *in vitro* and *in vivo* without obvious on-target off-tumor toxicities (16). Moreover, our clinical trial phase I study (NCT03932565) has been ongoing to examine the safety and feasibility of NECTIN-4-7.19 CAR-T cells in patients with NECTIN-4-positive malignant solid tumors.

CAR-T therapy for malignant solid tumors remains challenging owing to tremendous phenotypic heterogeneity, inefficient proliferation and short persistence of CAR-T cells, and immunosuppressive microenvironment in tumor stroma where inhibitory checkpoints lead to T-cell dysfunction, factors like adenosine and reactive oxygen species inhibit T cells, immunosuppressive cells like regulatory T cells and myeloid-derived suppressor cells promote tumor growth and inhibit T-cell activity, and cancer-associated fibroblasts deposit extracellular matrix to limit T cell penetration and recruit other immunosuppressive cells (16, 17). Co-stimulation is crucial in the development of effective adoptive immunotherapy of cancer (18–21). Many tumor cells not only down-regulate the expression of HLA (22, 23), but also down-regulate the ligand of costimulatory molecules to induce immune escape (24). The first-generation CAR T cells which without costimulatory molecules had very limited persistence and anti-tumor efficacy *in vivo* (25), and thus no real clinical efficacy (26). The second-generation CAR-T cells have enhanced tumor suppression ability and persistence given the addition of CD28 or 4-1BB co-stimulatory molecules (27, 28). To further enhance anti-tumor efficacy of TAC-T cells in the treatment of solid tumors, here we report a novel NECTIN-4-redirected TAC containing the co-stimulatory CD28 cytoplasmic domain. Our results showed that the co-stimulatory CD28 cytoplasmic domain could accelerate the proliferation of NECTIN-4 TAC28-T cells and enhance their cytotoxicity on target cells, and more importantly, increase the infiltration of NECTIN-4 TAC28-T cells into tumor lesions. Therefore, NECTIN-4 TAC28-T cells may be a safe and efficient treatment for solid tumors over-expressing NECTIN-4.

## 2 Materials and methods

### 2.1 Vector generation and lentiviral production

Lentiviral vectors encoding NECTIN-4 TAC, NECTIN-4 TAC28 and NECTIN-4 TAC28m were constructed by use of plenti-EF1α-MCS. NECTIN-4 TAC consisted of a single-chain variable fragment (scFv) derived from an antibody (16, 29) against human NECTIN-4, a single-chain variable fragment (scFv) derived from an antibody (30, 31) against human CD3ϵ, and a human CD4 domain (NP_001181944.1) (97-181aa), while NECTIN-4 TAC28 replaced CD4 cytoplasmic domain (145-181aa) with CD28 cytoplasmic domain (NP_006130.1) (179-220aa). And NECTIN-4 TAC28m was mutated with CD28 cytoplasmic domain (YMNM-YMFM, PRRP-ARRA) on the basis of NECTIN-4 TAC28. The cDNA sequence encoding these three TACs were codon-optimized, synthesized by GENEVA (Suzhou, China), and then cloned into plenti-EF1α-MCS. All these NECTIN-4 TAC lentiviruses were packaged in HEK-293T cells (ATCC, Manassas, VA, USA) by use of a third-generation lentivirus packaging system (pLP1, pLP2, and pMD2.G).

### 2.2 Cell lines

The human cell lines HEK-293T, MCF-7, MDA-MB-231 and ABC-1 was purchased from the American Type Culture Collection. ABC-1, MCF-7 and MDA-MB-231 cells were transduced with the lentivirus encoding the Firefly-Luciferase-GFP gene to generate ABC-1-Luc, MCF-7-Luc and MDA-MB-231-Luc; MDA-MB-231 cells were transduced with the lentivirus encoding the hNECTIN-4-Firefly-Luciferase-GFP gene to generate MDA-MB-231-hNECTIN-4-Luc. All cells were maintained in DMEM supplemented with 10% FBS in 5% CO_2_ at 37°C.

### 2.3 Generation of NECTIN-4-redirected TAC-T cells containing the co-stimulatory CD28 cytoplasmic domain

Primary T cells were isolated and activated from peripheral blood mononuclear cells (PBMC) of healthy volunteers by coating CD3/CD28 antibody magnetic beads (11141D, Gibco) with the ratio of T cells to anti-CD3/CD28 beads is 1:1.T cells were cultured in the media containing 50ng/mL IL-2, 12.5ng/mL IL-7, 12.5ng/mL IL-15 (Proteintech)at a culture concentration of 1 million/mL. 24-48 hours after T cell activation, T cells were transduced with NECTIN-4 TAC, NECTIN-4 TAC and NECTIN-4 TAC28m lentiviruses, respectively. And then the transduction efficiency was detected on day 5.

### 2.4 Flow cytometry

The expression of NECTIN-4 on the surface of tumor cells was detected by anti-human NECTIN-4 Alexa Fluor^®^ 647-conjugated antibody (R&D Systems, USA); The expression of NECTIN-4 TAC, NECTIN-4 TAC28 and NECTIN-4 TAC28m was detected by the fusion protein of NECTIN-4 extracellular domain labelled with Biotin (Acro), and then followed by the PE/APC streptavidin (Biolegend).

TAC-T cell phenotypes were assessed with monoclonal antibodies against the following molecules: CD4 (FITC Biolegend), CD8 (Pecy7/PE Biolegend), CCR7 (FITC Biolegend), CD45RA (APC Biolegend), CD27 (APC Biolegend), CD28 (Pecy7 Biolegend). TAC-T cell activation level was assessed with monoclonal antibodies against the following molecules CD25 (FITC Biolegend) and CD69 (Pecy7 Biolegend). For analysis of immunological checkpoints, the following antibodies were used: PD1 (PE Biolegend), TIM3 (FITC Biolegend) and LAG3 (PE Biolegend). Cells were analyzed by a FACS Aria IIFlow Cytometer (BD Biosciences). Data were analyzed with FlowJo 10 (FlowJo, USA).

### 2.5 Cytokine secretion analysis

Enzyme-linked immunosorbent assay (ELISA) was used to quantify the concentration of cytokines and chemokines. Culture supernatants of TAC-T cells were collected and then detected by an INF-γ,TNF-α and IL-2 ELISA kit.

### 2.6 Proliferation analysis

TAC-T cells were labeled with Cell Trace™ CFSE (Thermo Fisher Scientific) and co-cultured with NECTIN-4-beads at an Effect/Target ratio of 1:1 in a 24-well plate without the addition of external cytokines for 5 days and then analyzed by use of a flow cytometer with 488-nm excitation and emission filters appropriate for fluorescein to evaluate the proliferation of TAC-T cells.

Short-term proliferation of TAC-T cells co-cultured with NECTIN-4-beads was assessed with the CCK8(Sigma-Aldrich) as follows. TAC-T cells and NECTIN-4-beads were cultured in a 96-well plate at a 1:1 ratio and cocultured in the absence of IL-2 for 3 days. At the end of the experiments, CCK8 solution was added into each well and the cells were further incubated at 37 °C for 3 h. The optical density was measured at 450 nm using a spectrophotometer.

### 2.7 Cytotoxicity analysis

The xCELLigence RTCA MP instrument (Acea Biosciences Inc, CA, USA) was utilized for the assessment of TAC-T cell-mediated cytotoxicity. Briefly, 1x10^4^ tumor cells were seeded on each well of an E-Plate 16 (Acea Biosciences) and grew until their adherence. Then, TAC-T cells were added into each unit at different Effect/Target ratios, with media or 2.5% Triton-X 100 (Solarbio, Beijing, CN) as negative or positive controls. Each group consisted of three replicate wells and the impedance signals (Cell index) were recorded for a duration of 0-80 h. Electrical impedance was quantified every 15 min by the use of the RTCA DP Analyzer.

The target cells expressing luciferase were cultured in a 96-well culture plate one day in advance, and the target cells were lysed with different ratios of effector cells and target cells on the second day, and MOCK-T cells were used as controls. The culture wells of target cells without effector cells were used as the negative controls (Kmax), and the culture wells of target cells added with ddH2O were used as the positive control (Kmin). After 24 hours, 0.5 mM D-luciferin (MedChemExpress, Shanghai, CN) was added to each experimental well and the fluorescence intensity value (K) was detected with a microplate reader, and the cell lysis efficiency was equal to (K_min_-K)/(K_min_-K_max_)x100%.

### 2.8 Animal experiments

NSG mice aged 4-6 weeks were subcutaneously inoculated with NECTIN-4-MDA-MB-231-luc-GFP cells, and the tumors were observed by IVIS imaging system 7 days later. Mice were randomly divided into the four groups. The MOCK-T cells, NECTIN-4 TAC-T cells, NECTIN-4 TAC28-T cells and NECTIN-4 TAC28m-T cells were injected into the mice through the tail vein with the MOCK-T cell injection group used as the negative control. Tumor growth in mice was regularly observed by IVIS imaging.

### 2.9 RNA-seq

NECTIN-4 TAC-T and NECTIN-4 TAC28-T cells sorted by flow cytometry were incubated with NECTIN-4-beads for 24 hours. Then, the cells were washed with PBS twice, and directly sorted into Trizol and stored at -80°C. The samples were sent to Jinweizhi (Suzhou, China) for RNA sequencing.

### 2.10 Statistical analysis

Data were analyzed as mean ± SD by t-test. Survival curve was analyzed by Kaplan–Meier curves and log-rank test. p-values < 0.05 were considered statistically significant. All experiments were repeated at least three times. All statistical analyses were performed with GraphPad Prism v9.0 (GraphPad Prism, USA).

## 3 Results

### 3.1 NECTIN-4 TAC28-T cells showed no tonic signal

**Figure 1:**
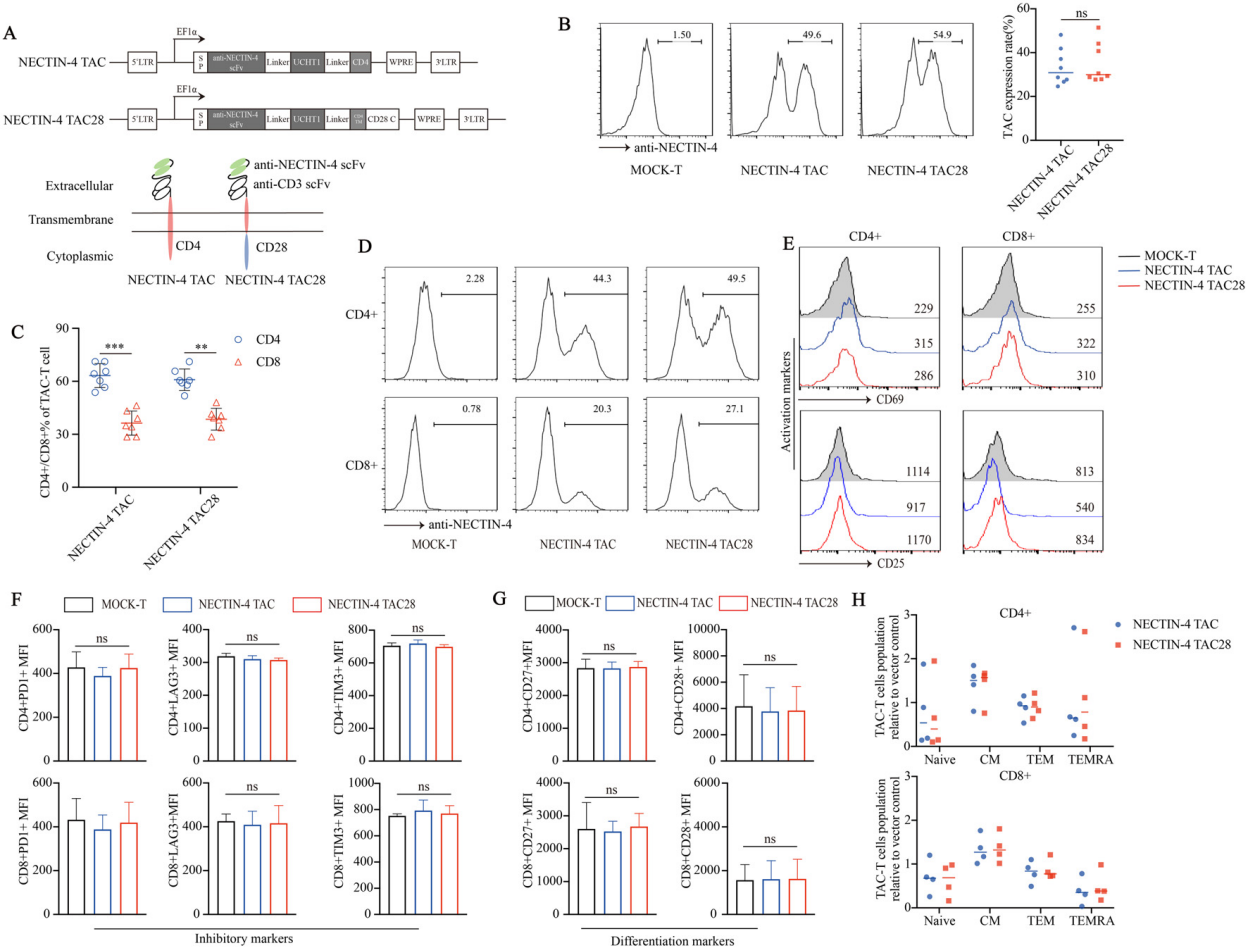
Successful generation of CD28-containing NECTIN-4 TAC28-T cells without tonic signal. **(A)** Schematic illustration of NECTIN-4 TAC and NECTIN-4 TAC28 constructs. **(B)** TAC expression in NECTIN-4 TAC-T and NECTIN-4 TAC28-T was detected by flow cytometry. **(C)** The ratio of CD4^+^ and CD8^+^ T cells of TAC-T cells. **(D)** The expression of TAC in CD4^+^ and CD8^+^ T cells. **(E)** CD69 and CD25 expression. **(F)** Expression of checkpoint receptors PD-1, LAG-3, and TIM-3. **(G)** CD27 and CD28 expression. **(H)** Memory T cells subsets of NECTIN-4 TAC-T and NECTIN-4 TAC28-T cells relative to MOCK-T cells. T cell subsets are defined as naïve (CD45RA^+^, CCR7^+^), central memory (CD45RA^-^, CCR7^+^), effector memory (CD45RA^-^, CCR7^-^), and terminal effectors (CD45RA^+^, CCR7^-^). For **(E–G)**, the histograms represented the data from three healthy donors, and median fluorescence intensity was indicated. ns, no significant difference, **p < 0.01, ***p < 0.001, t-test. Data are presented as the mean ± SD.

We prepared two kinds of engineered T cells targeting NECTIN-4, namely NECTIN-4 TAC-T cells and NECTIN-4 TAC28-T cells. Different from the intracellular CD4 domain of NECTIN-4 TAC-T cells, that of NECTIN-4 TAC28-T cells is co-stimulatory CD28 cytoplasmic domain (**Figure 1A**). Anti-NECTIN-4 scFv expression was assessed to be 33.48 ± 8.273% and 35.06 ± 9.087% in NECTIN-4 TAC-T cells and NECTIN-4 TAC28-T cells respectively (*p*=0.7203) (**Figure 1B**), and the proportion of CD4^+^ T cells was higher than that of CD8^+^ T cells in both anti-NECTIN-4 engineered T cells (**Figure 1C**). Lentiviruses infected activated CD4^+^ and CD8^+^ T cells at the same Multiplicity of infection (MOI), and CD4^+^ T cells were more likely to express NECTIN-4 TAC and NECTIN-4 TAC28 (**Figure 1D**). Tonic signaling of CAR-T cells, i.e, spontaneous activation of CAR signaling in the absence of tumor antigen stimulation, is considered to be one of the factors affecting CAR-T cells persistence and differentiation. TAC-T cells utilize endogenous TCR so as not to induce tonic signaling. To verify that incorporation of the co-stimulatory CD28 cytoplasmic domain does not induce the tonic signaling of NECTIN-4 TAC28-T cells, we measured CD25 and CD69 expression levels of MOCK-T, NECTIN-4 TAC-T, and NECTIN-4 TAC28-T cells, the well-defined biomarkers of T cell activation used to indicate the T cell tonic signaling (**Figure 1E**). Furthermore, we examined exhaustion biomarkers including PD1, Tim3 andLAG3, and T cell subsets defined by CCR7, CD45RA (**Figures 1F–H**). The results showed that there was no significant difference between NECTIN-4 TAC28-T cells and NECTIN-4 TAC-T cells.

### 3.2 The incorporated CD28 cytoplasmic domain accelerated the activation and proliferation of NECTIN-4 TAC28-T cells

**Figure 2:**
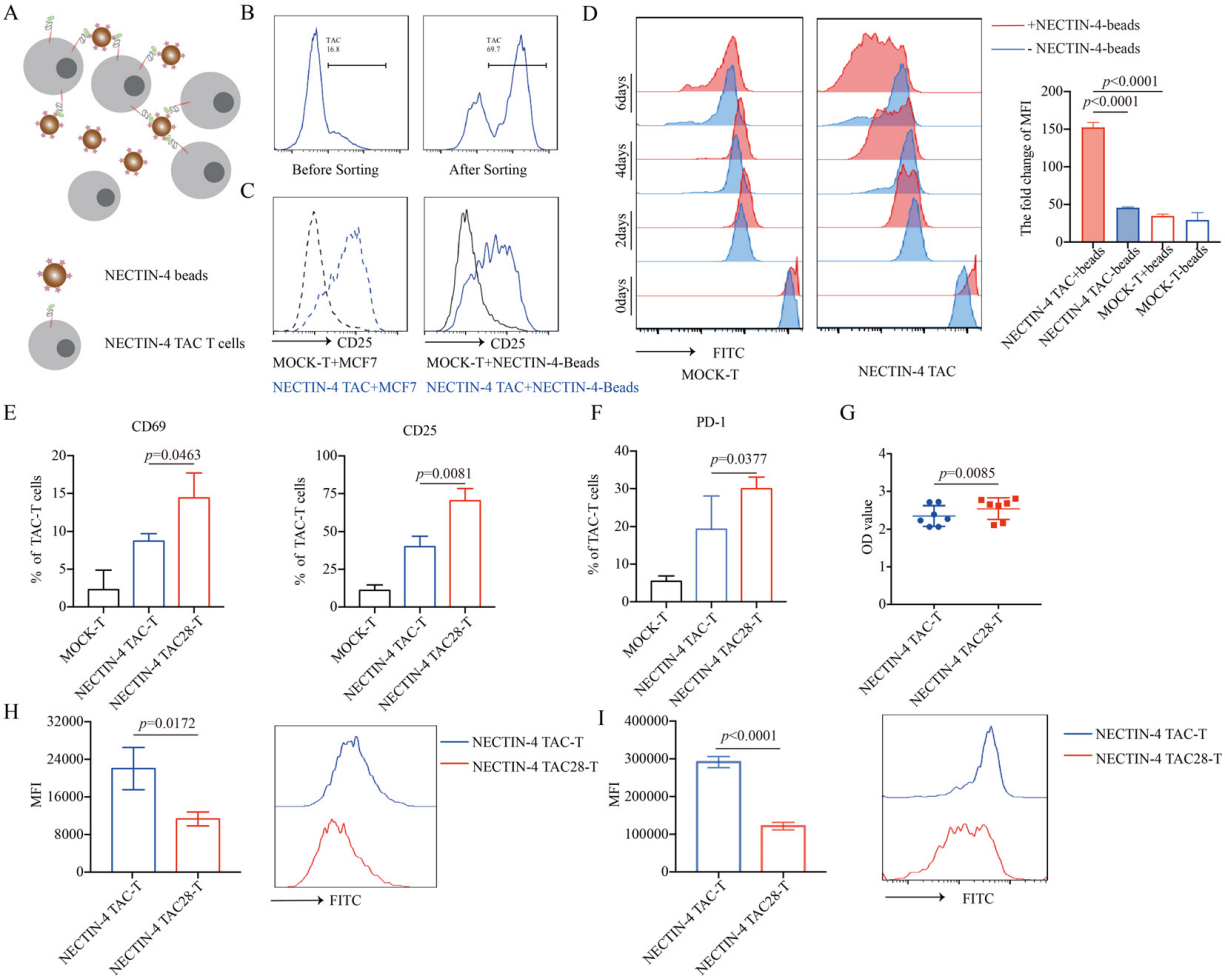
NECTIN-4 TAC28-T cells showed improved activation and proliferation. **(A)** Schematic diagram of NECTIN-4-beads interacted with anti-NECTIN-4 TAC-T cells. **(B)** TAC expression after NECTIN-4-beads sorting. **(C)** CD25 expression of MOCK-T and NECTIN-4-redirected TAC-T cells upon stimulation with MCF-7 cells or NECTIN-4-beads. **(D)** MOCK-T and NECTIN-4-redirected TAC-T cells were labeled with carboxyfluorescein succinimidyl ester (CFSE), and their proliferation were assessed by flow cytometry upon stimulation with NECTIN-4-beads.The left side is the flow cytometry graph, and the right side is the ratio change of cell surface CFSE fluorescence values on day 0 and day 6. **(E)** CD69 and CD25 expression of MOCK-T, NECTIN-4 TAC-T and NECTIN-4 TAC28-T cells upon stimulation with NECTIN-4-beads. **(F)** The expression of PD-1 on TAC-T cells. **(G)** NECTIN-4 TAC-T and NECTIN-4 TAC28-T cells were stimulated by NECTIN-4-beads, and their proliferation were assessed through cell counting by use of CCK8 method. **(H)** NECTIN-4 TAC-T and NECTIN-4 TAC28-T cells were labeled with CFSE, and their proliferation were assessed by flow cytometry upon stimulation with NECTIN-4-beads. **(I)** NECTIN-4 TAC and NECTIN-4 TAC28 CD4^+^ T cells were labeled with CFSE, and their proliferation were assessed by flow cytometry upon stimulation with NECTIN-4-beads. Data came from ≥3 donors. ns, no significant difference, t-test. Data were presented as the mean ± SD.

Specific antigens can activate and proliferate CAR-T cells, magnetic beads coated with NECTIN-4 proteins (NECTIN-4-beads) were used as antigens to stimulate anti-NECTIN-4 TAC-T cells. First, we demonstrated that NECTIN-4-beads could bind and purify NECTIN-4 TAC-T cells (**Figures 2A, B**). After co-incubating NECTIN-4 TAC-T cells with NECTIN-4-beads for 24 hours, we observed that CD25 was highly expressed on the surface of NECTIN-4 TAC-T cells, while NECTIN-4^+^ MCF-7 cells were used as a positive control (**Figure 2C**). CFSE-labeled MOCK-T cells and NECTIN-4 TAC-T cells were co-incubated with NECTIN-4-beads, and the proliferation was detected on days 2, 4, and 6 by FACS, respectively. We found that NECTIN-4-beads could specifically expand NECTIN-4 TAC-T cells (**Figure 2D**). The above results indicated that NECTIN-4-beads could be used as the specific antigen to analyze the difference between NECTIN-4 TAC-T cells and NECTIN-4 TAC28-T cells. CD28 can accelerate the proliferation and rapid activation of CAR-T cells (32). Stimulated by NECTIN-4-beads for 24 hours, NECTIN-4 TAC-T cells and NECTIN-4 TAC28-T cells expressed CD69 (8.72 ± 0.9924% *vs* 14.4 ± 3.305%; *p*=0.0463), CD25 (40 ± 6.991% *vs* 70.27 ± 8.133; *p*=0.0081), PD1 (19.33 ± 8.722% *vs* 29.98 ± 3.177%; *p*=0.0377) (**Figures 2E, F**). We further demonstrated that CD28 could accelerate the proliferation of NECTIN-4 TAC28-T cells with CCK8 and CFSE methods, respectively (**Figures 2G, H**). Moreover, we labeled the CD4^+^ NECTIN-4-redirected TAC-T cells with CFSE and found that the MFI on the surface of CD4^+^ NECTIN-4 TAC28-T cells was 121351 ± 9992 as opposed to 291348 ± 8553 for NECTIN-4 TAC-T cells after 72 h of culture (**Figure 2I**). Besides, we added CD28 cytoplasmic domain after CD4 cytoplasmic domain, and constructed NECTIN-4 TAC4 + 28 vector (**Supplementary Figure 2A**). Unexpected, NECTIN-4 TAC4 + 28-T cells didn’t exhibit stronger early activation and proliferation ability than NECTIN-4 TAC-T cells (**Supplementary Figures 2B, C**). We also replaced CD4 cytoplasmic domain with 41BB cytoplasmic domain, and thus constructed NECTIN-4 TAC41BB vector (**Supplementary Figure 2A**). However, compared with NECTIN-4 TAC-T cells, NECTIN-4 TAC41BB-T cells displayed lower activation level and slower proliferation rate when co-cultured with NECTIN-4-beads (**Supplementary Figures 2D, E**).

### 3.3 Incorporated CD28 cytoplasmic domain enhanced the cytotoxicity of NECTIN-4 TAC28-T cells on target cells

**Figure 3:**
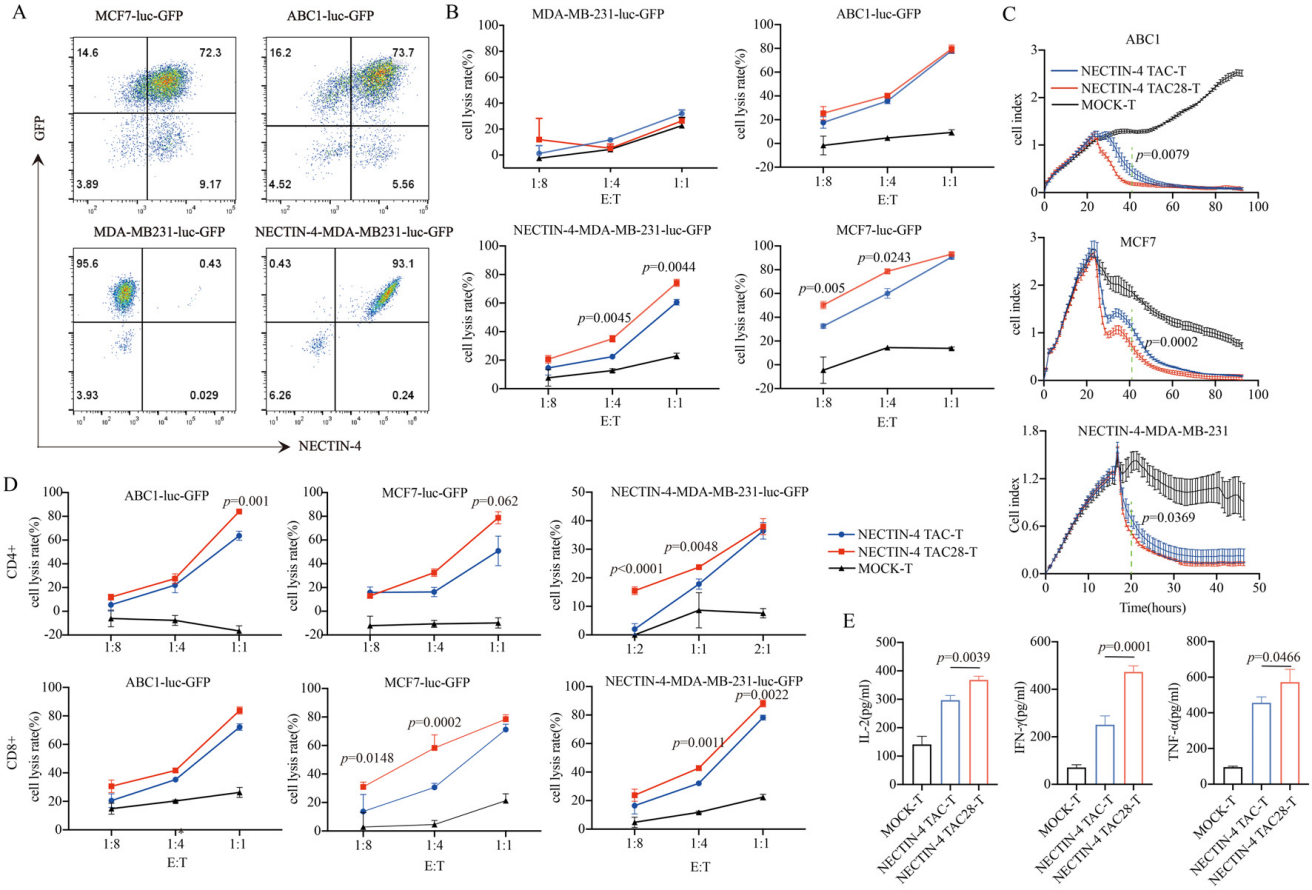
Specific target cell lysis by NECTIN-4-redirected T cells. **(A)** NECTIN-4 and GFP expression in MCF7-luc-GFP, ABC1-luc-GFP, MDA-MB-231-luc-GFP and NECTIN-4-MDA-MB-231-luc-GFP cells transduced with the lentivirus encoding the Luciferase-T2A-GFP or NECTIN-4-P2A-Luciferase-T2A-GFP. **(B)** Cytotoxicity of NECTIN-4 TAC-T and NECTIN-4 TAC28-T cells were assessed by co-incubation with luciferase-expressing MCF7-luc-GFP, ABC1-luc-GFP, MDA-MB-231-luc-GFP and NECTIN-4-MDA-MB-231-luc-GFP cells at the indicated E/T ratio. **(C)** Real-time analysis (RTCA) was used to monitor the cytolysis of ABC1, MCF7 and MB231-NECTIN-4 cells by NECTIN-4 TAC-T and NECTIN-4 TAC28-T cells. Effector: target (E:T) cell ratio=1:1. **(D)** Cytotoxicity of NECTIN-4 TAC and NECTIN-4 TAC28 CD4^+^/CD8^+^ cells were assessed by co-incubation with luciferase-expressing NECTIN-4-MDA-MB-231-luc-GFP, MCF7-luc-GFP and ABC1-luc-GFP at the indicated E/T ratio. **(E)** ELISA was used to detect the secretion of IL-2, IFN-γ, and TNF-α by NECTIN-4-redirected TAC-T cells after co-culture with MCF7 cells for 24 h. Data came from ≥3 donors. ns, no significant difference, t-test. Data are presented as the mean ± SD.

NECTIN-4 expression of breast cancer cell lines MCF7, MDA-MB-231 and lung cancer cell line ABC1 were detected by flow cytometry. Both MCF7 and ABC1 over-expressed NECTIN-4, while the MDA-MB-231 expressed no NECTIN-4 (**Supplementary Figure 1**). To verify the specific cytotoxicity of NECTIN-4 TAC28-T cells, we constructed MDA-MB-MB231, MCF7 and ABC1 cell lines to express both luciferase and GFP. Further, we constructed the NECTIN-4-MDA-MB-231cell line to over-express NECTIN-4 as a positive control (**Figure 3A**). The cytotoxicity of NECTIN-4-redirected TAC-T cells towards MDA-MB-MB231, NECTIN-4-MDA-MB-MB231, MCF7 and ABC1 was assessed by luciferase assay at an effector to target (E:T) ratio of 1:8 to 1:1. NECTIN-4 TAC28-T cells exhibited strong cytotoxicity on NECTIN-4-MDA-MB-MB231 cells but not MDA-MB-MB231 cells (**Figure 3B**). Accordingly, NECTIN-4 TAC28-T cells had a better ability to lyse target cells *in vitro* by RTCA method (**Figure 3C**). We further found that CD4^+^ NECTIN-4 TAC28-T cells and CD8^+^ NECTIN-4 TAC28-T cells lysed target cells stronger than CD4^+^ NECTIN-4 TAC-T cells and CD8^+^ NECTIN-4 TAC-T cells, respectively (**Figure 3D**). Compared with NECTIN-4 TAC-T cells, NECTIN-4 TAC4 + 28-T cells did not show superior cytotoxicity against target cells. For example, NECTIN-4 TAC-T cells showed better lysing ability than NECTIN-4 TAC4 + 28-T cells in term of lysis of ABC1 at an effector-target ratio of 1:1 (**Supplementary Figure 3A**). Unexpectedly, NECTIN-4 TAC41BB-T cells exhibited worse cytotoxicity than NECTIN-4 TAC-T cells against target cells (**Supplementary Figure 3B**). Comparing IL-2, IFN-γ and TNF-α secretion between NECTIN-4 TAC-T and NECTIN-4 TAC28-T cells after 24-h incubation with MCF7 target cells (E:T=1:1), NECTIN-4 TAC28-T cells were found to secrete more IL-2, IFN-γ and TNF-α than NECTIN-4 TAC-T cells (368.1 ± 13.05 pg/ml *vs* 297 ± 15.94 pg/ml, p=0.0039; 250.7 ± 37.73 pg/ml *vs* 473.4 ± 25.30 pg/ml, p=0.0001; and 456.6 ± 32.36 pg/ml *vs* 572.4 ± 71.47pg/ml, p=0.0466; respectively) (**Figure 3E**).

### 3.4 NECTIN-4 TAC28-T had a unique transcriptional profile upon stimulation with NECTIN-4-beads

**Figure 4:**
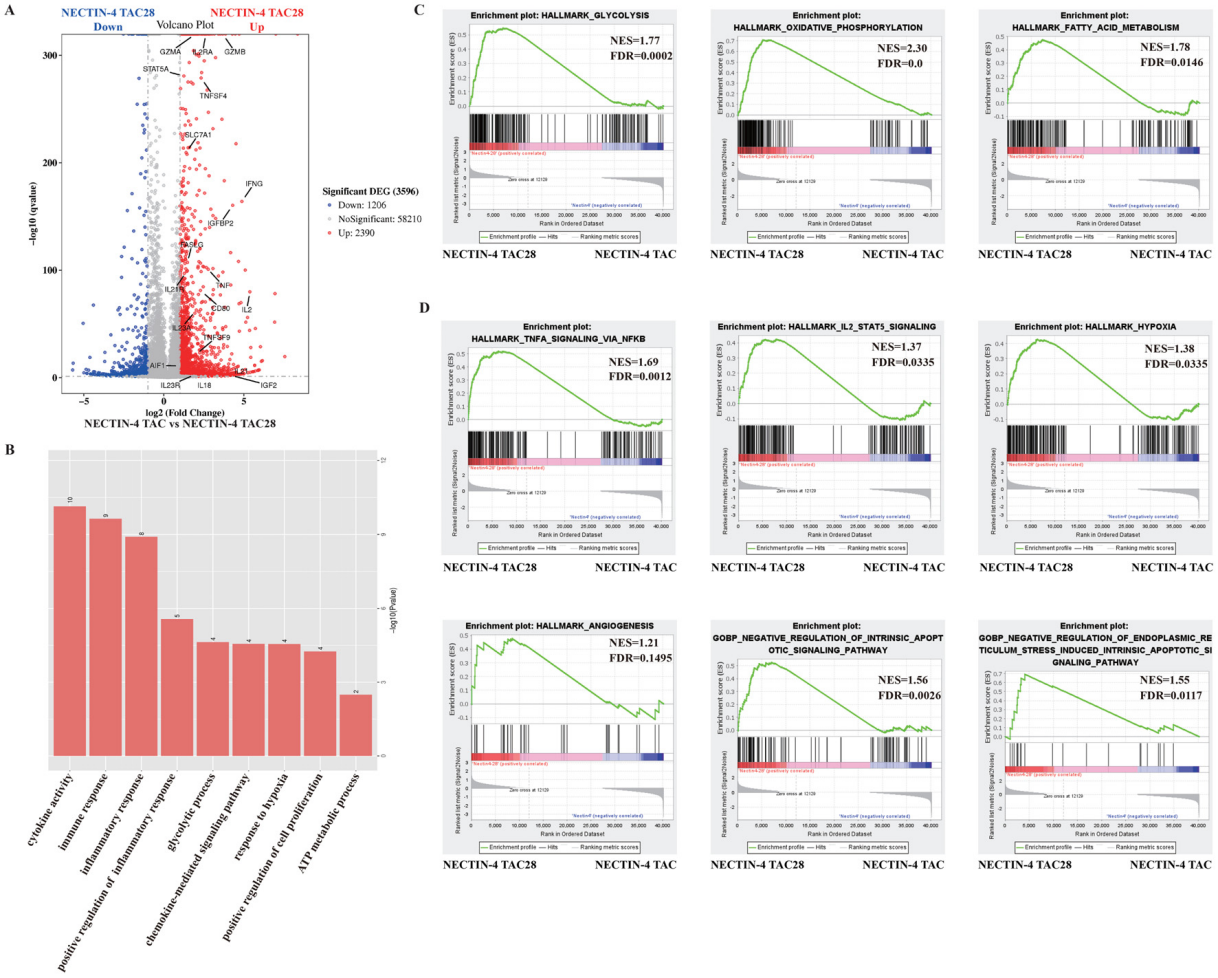
The effects of incorporated CD28 cytoplasmic domain on the transcriptional profile of NECTIN-4 TAC28-T cells upon stimulation with NECTIN-4-beads. **(A)** Differentially expressed genes between NECTIN-4 TAC-T and NECTIN-4 TAC28-T cells. **(B)** Gene Ontology analysis of the different genes between NECTIN-4 TAC-T and NECTIN-4 TAC28-T cells. **(C, D)** Representative GSEA results from running the unfiltered NECTIN-4 TAC-T versus NECTIN-4 TAC28-T cell rank list against the MSigDB H hallmark gene sets and C5 gene ontology sets.

We next sought to identify molecular pathways that contribute to the improving effects of the incorporated CD28 cytoplasmic domain on NECTIN-4 TAC28-T cell function. Global transcriptional profiles of MOCK-T, NECTIN-4 TAC-T and NECTIN-4 TAC28-T cells from three donors were analyzed 9 days after initial *in vitro* activation. NECTIN-4 TAC28-T cells showed higher expression of several genes associated with T cell activation and effector function including IL2RA, GZMB, GZMA, IFNG, TNF, IL21R and FASLG, indicating NECTIN-4 TAC28-T cells were more strongly activated when stimulated by NECTIN-4 antigen than NECTIN-4 TAC-T cells. Moreover, the expression of the genes associated with T cells proliferation (i.e.,IL-2, IL23A, IGFBP2, TNFSF9, IL23R, IL18) were up-regulated in NECTIN-4 TAC28-T cells (**Figure 4A**). The gene ontology analysis showed that there were a lot of biological process (i.e., positive regulation of cell proliferation, immune response, response to hypoxia, glycolytic process) differences between NECTIN-4 TAC-T and NECTIN-4 TAC28-T cells (**Figure 4B**).

We also conducted unbiased comparisons of NECTIN-4 TAC-T versus NECTIN-4 TAC28-T cells to identify the pathways that might contribute to the improved functionality observed in NECTIN-4 TAC-28 T cells. Gene Set Enrichment Analysis (GSEA) with the Molecular Signatures Database identified numerous gene sets enriched in NECTIN-4 TAC-28 T cells that were associated with energy metabolism including glycolysis, fatty acid metabolism and oxidative phosphorylation (**Figure 4C**, **Supplementary Figure 4**). Other enriched gene sets included TNFA signaling, IL-2 STAT5 signaling, hypoxia, angiogenesis and negative regulation of apoptotic signaling, which may play important roles for TAC-T cells in lysing tumor cells and surviving in the immunosuppressive microenvironment (**Figure 4D**).

### 3.5 Mutations in the incorporated CD28 cytoplasmic domain attenuated the proliferation and activation of NECTIN-4 TAC28-T cells

**Figure 5:**
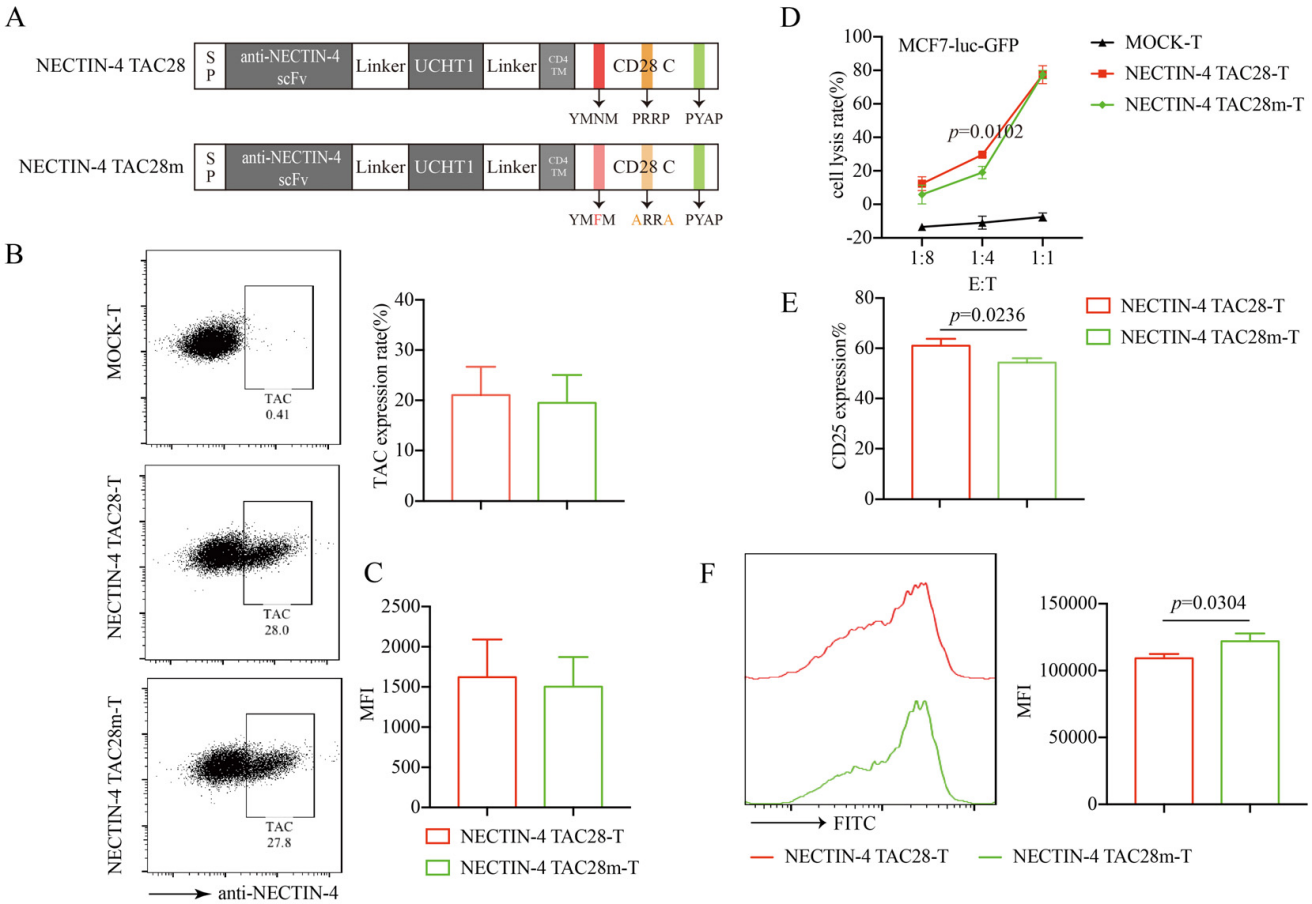
Effects of mutations in the incorporated CD28 cytoplasmic domain on NECTIN-4 TAC28-T function. **(A)** Schematic illustration of NECTIN-4 TAC28m construct design. **(B)** TAC expression in NECTIN-4 TAC28-T and NECTIN-4 TAC28m-T was detected by flow cytometry **(C)** MFI of anti-NECTIN-4 scFv on the surface of NECTIN-4-redirected TAC-T cells. **(D)** Cytotoxicity of NECTIN-4 TAC28-T and NECTIN-4 TAC28-T cells were assessed by co-incubation with luciferase-expressing MCF7-Luc cells at the indicated E/T ratio. **(E)** CD25 expression of NECTIN-4-redirected TAC-T cells was detected by flow cytometry. **(F)** NECTIN-4 TAC28-T and NECTIN-4 TAC28m-T cells were labeled with CFSE, and their proliferation was assessed by flow cytometry after simulation with NECTIN-4-beads. Data came from ≥3 donors. ns, no significant difference, t-test. Data are presented as the mean ± SD.

CD28 cytoplasmic domain consists of YMNM, PRRP, and PYAP subdomains that regulate signaling pathways following TCR stimulation (33). To verify that the incorporated CD28 cytoplasmic domain enhanced the activation and proliferation of NECTIN-4 TAC28-T cells, we mutated 2 intracellular subdomains in CD28 (YMNM→YMFM, PRRP→ARRA) (**Figure 5A**). We confirmed these mutations did not alter the expression or mean fluorescence intensity of anti-NECTIN-4 scFv on the surface of T cells (**Figures 5B, C**). We also evaluated NECTIN-4 TAC28m-T cell cytotoxicity by use of luciferase assay and determined that at a low (1:4) effector-to-target (E:T) ratio, NECTIN-4 TAC28m-T cells showed lower cytotoxicity than NECTIN-4 TAC28-T cells (**Figure 5D**). Moreover, compared with NECTIN-4 TAC28-T cells, the expression level of CD25 in NECTIN-4 TAC28m-T cells stimulated by NECTIN-4-beads was lower (61 ± 2.771% *vs* 54.27 ± 1.750; p=0.0236) (**Figure 5E**). In addition, we found NECTIN-4 TAC28m-T cells proliferated much slower than NECTIN-4 TAC28-T cells (**Figure 5F**). These results demonstrated that the stronger activation, faster proliferation and better cytotoxicity of NECTIN-4 TAC28-T cells could be attributed to the incorporated CD28 cytoplasmic domain.

### 3.6 NECTIN-4 TAC28-T cells induced persistent tumor regression and long-term remission *in vivo*

**Figure 6:**
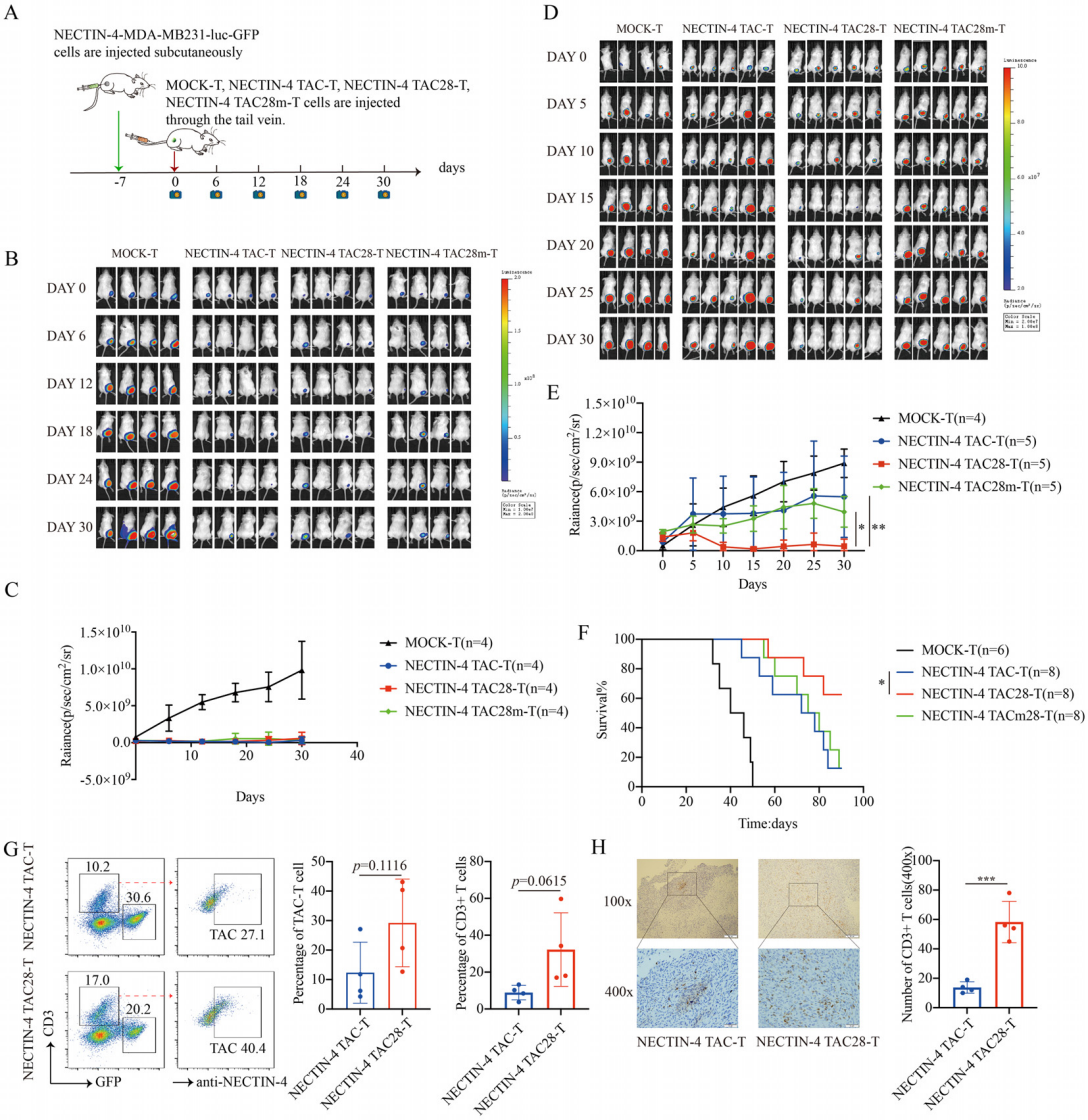
NECTIN-4 TAC28-T cells demonstrated improved efficacy over NECTIN-4 TAC-T and NECTIN-4 TAC28m-T cells *in vivo*. **(A)** Treatment scheme for NECTIN-4-MDA-MB-231-luc-GFP tumor-bearing mice. **(B-E)** Tumor xenografts were monitored via bioluminescence imaging. Bioluminescence images and kinetics were shown in **(B, C)** at E/T=3:1(3million:1million); as well as in **(D, E)** at E/T=1:1(1million:1million). **(F)** Kaplan–Meier survival curve, p=0.0471. **(G)** Proportion of NECTIN-4 TAC-T, NECTIN-4 TAC28-T and NECTIN-4-MDA-MB-231-luc-GFP cells in tumor lesions, detected by flow cytometry (n=4). **(H)** The infiltration of CD3^+^ T cells inside the tumor lesions of both the NECTIN-4 TAC-T cell treatment group and the NECTIN-4 TAC28-T cell treatment group was analyzed by immunohistochemistry (n=4). ns, no significant difference, *p < 0.05, **p < 0.01, ***p < 0.001, One-way ANOVA-test. Data are presented as the mean ± SD. These data are representative of three independent experiments.

To assess the antitumor efficacy of NECTIN-4-redirected TAC-T cells in an *in vivo* model, NSG mice were inoculated with NECTIN-4-MDA-MB-231-luc-GFP cells, followed by NECTIN-4 TAC, NECTIN-4 TAC28-T or NECTIN-4 TAC28m-T cells treatment 7 days post tumor engraftment (**Figure 6A**). Mice treated with 3 million NECTIN-4 TAC-T, NECTIN-4 TAC28-T or NECTIN-4 TAC28m-T cells exhibited significant reduction in tumor burden as compared with control mice, but there was no significant difference among the three treatment groups (**Figures 6B, C**). However, when the number of treated cells was reduced to 1 million, tumor growth was more significantly delayed and the survival was prolonged in NECTIN-4 TAC28 group compared with NECTIN-4 TAC and NECTIN-4 TAC28m groups (**Figures 6D–F**). We further evaluated murine body weight and vital organs after treatment, and found that incorporated CD28 cytoplasmic domain did not impair the safety of NECTIN-4 TAC28-T cell therapy (**Supplementary Figures 5A, B**). Furthermore, to compare the difference in tumor infiltration between NECTIN-4 TAC-T cells and NECTIN-4 TAC28-T cells, we established a subcutaneous tumor mouse model. Mice was treated with 1 million NECTIN-4 TAC-T cells or NECTIN-4TAC28-T cells. 10 days later, tumor tissues were collected for flow cytometry and histochemical staining to analyze T cell infiltration. We found that the proportions of T cells in the NECTIN-4 TAC28-T and NECTIN-4 TAC-T treatment group were 32.23 ± 19.94 and 8.895 ± 3.951, respectively (p=0.0615) (**Figure 6G**). In line with the observation above, immunohistochemistry results showed that there were more infiltrating T cells in the TAC28-T group than in the TAC-T group (13.75 ± 3.862 *vs* 58.25 ± 14.01;p=0.0009) (**Figure 6H**). In addition, we constructed a mouse metastatic tumor model using NECTIN-4-MDA-MB-231-luc-GFP cells and demonstrated that NECTIN-4 TAC28-T cells could more effectively inhibit tumor growth compared with NECTIN-4 TAC-T cells(**Supplementary Figure 6**).

## 4 Discussion

CAR-T cells therapy is well-known for its outstanding therapeutic efficacy in hematological tumors. Six CAR-T cell therapies have been approved by the FDA, but they still face challenges such as severe toxic side effects and poor therapeutic effect on solid tumors (3, 17, 34, 35). TAC-T cells can induce more effective antitumor responses and reduce toxicity through endogenous TCR (11). Tonic signaling induces CAR-T cell dysfunction, and thus transient mTOR inhibition of tonic signaling can reverse the function of 4-1BB CAR-Tregs cells (36). TAC-T cells don’t elicit tonic signaling (37). NECTIN-4 TAC28-T cells did not induce cell activation without antigen stimulation *in vitro*, and neither accelerated T cell differentiation, nor up-regulated exhaustion markers such as PD1, LAG3, and TIM3 expression. It is well known that the subtype and differentiation status of T cells are crucial for the efficacy of CAR-T cells (38).

NECTIN-4 TAC28-T cells displayed faster and higher cell-surface levels of some T cell activation biomarkers, including CD25 and CD69, indicating a more efficient activation of NECTIN-4 TAC28-T cells upon antigen stimulation. However, there were no significant differences of anti-tumor ability between NECTIN-4 TAC4 + 28-T cells and NECTIN-4 TAC-T cells, implying that the position of CD28 is important for NECTIN-4 TAC-T cells. Unlike NECTIN-4 TAC28-T cells, the anti-tumor ability of NECTIN-4 TAC41BB-T cells was not enhanced. The results indicated the CD28 domain was able to link with Lck which may be enough to substitute CD4 in NECTIN-4 TAC-T cells. IL-2 maintains the proliferation and differentiation of T cells (39). NECTIN-4 TAC28-T cells secreted much more IL-2, and thus transcriptome genes were enriched in the IL-2-STAT 5 signaling pathway, which was the direct evidence for the faster proliferation of NECTIN-4 TAC28-T cells. Furthermore, IL23A, IL23R (40), IL-18, IFN-γ (41), etc. were highly expressed in NECTIN-4 TAC28-T cells, which also confirmed that CD28 could promote the proliferation of NECTIN-4 TAC28-T cells. Activation is associated with a biosynthetic and bioenergetics flux required to support T cell proliferation and function (42, 43). Naïve and memory T cells rely primarily on the mitochondrial oxidation of fatty acids for development and persistence (44, 45). Activated effector T cells shift to glycolysis or concurrently upregulate oxidative phosphorylation and aerobic glycolysis to fulfill the metabolic demands of proliferation (45, 46). The second-generation CAR-T cells expressing the CD28 signaling domain have enhanced glycolysis and rapid antitumor effect, but exhibit poor persistence *in vivo* (47–49), and the 4-1BB signaling domain enables CAR-T cells to mainly use mitochondrial respiration and lipid oxidation to maintain cellular memory subtypes and prolong persistence (50). Some CAR-T cells harboring both CD28 and 4-1BB domains display rapid effector functions via glycolysis, but they also retain oxidative functions that support memory formation and long-term persistence (51, 52), suggesting that the combination of multiple metabolic pathways is suitable for T cells to exert anti-tumor effects. Our results demonstrated that NECTIN-4 TAC28-T cells upregulated glycolysis, fatty acids metabolism and oxidative phosphorylation pathways upon stimulation with NECTIN-4-beads. Unlike CAR-T cells, TAC-T cells transmit the first signal through TCR upon antigen stimulation. Integration of the CD28 co-stimulatory domain in NECTIN-4 TAC28-T cells could enhance not only glycolysis but also fatty acid metabolism and oxidative phosphorylation pathways. These results suggested that the metabolic pattern of NECTIN-4 TAC28-T cells may contribute to their persistence and anti-tumor functions.

CD28 co-stimulatory molecules include at least three subunits, YMNM, PRRP, and PYAP, which are involved in signal transmission (33). CD28 directly activates PI3K and Grb2 signaling pathways through YMNM (53, 54), and thus shorten terminal T cell persistence. Mutation of YMNM to YMFM enhances the persistence of CAR-T cells in tumors of xenografted mice (55). ITK can bind to PRRP motif, which signals through PLCγ and Erk, leading to T cell proliferation and IL-2 secretion (56). The PYAP motif initiates signaling by binding Lck in CAR-T cells (57), and the CD28 retaining only the distal PYAP subdomain can enhance the function and persistence of CAR-T cells *in vivo (* 33, 58, 59). In the structure of NECTIN-4 TAC28m, YMNM is mutated to YMFM, PRRP is mutated to ARRA, and the PYAP domain is retained. Unexpectedly, compared with the NECTIN-4 TAC-T treatment group, the NECTIN-4 TAC28m-T treatment group did not show better function *in vivo*, indicating that the CAR-T cell signaling may be different from TAC28-T cell signaling. *In vitro*, we observed that proliferation and cytotoxicity of NECTIN-4 TAC28m-T cells were impaired, further proving that the optimized function of NECTIN-4 TAC28-T cells could be attributed to the incorporated CD28 cytoplasmic domain.

These data highlight the importance of the co-stimulatory domain in the optimization of TAC-T cells, as the co-stimulatory domain in the second-generation CAR-T cells prolongs the persistence of CAR-T cells *in vivo* and enhances the cytotoxicity of CAR-T cells (60). When we treated NECTIN-4-expressing mouse xenograft tumor models with high dosage of NECTIN-4-redirected TAC-T cells, we found no significant difference. But when we decreased the dosage, NECTIN-4 TAC28-T cells still-effectively inhibited tumor growth. Importantly, more NECTIN-4 TAC28-T cells than NECTIN-4 TAC-T cells were infiltrated into the tumor lesions, which might reflect better migration capacity of NECTIN-4 TAC28-T cells into solid tumors and/or their superior expansion and persistence within the tumor.

In summary, we have constructed a novel optimized TAC-T cell (i.e., TAC28-T) with faster proliferation and stronger cytotoxicity, which not only contains CD28 co-stimulatory domain but also utilizes endogenous TCR to transmit T activation signals. The TAC28-T platform may represent a safe and highly effective therapeutic strategy, especially for the treatment of solid tumors.

## Data Availability

The authors acknowledge that the data presented in the study are publicly available at (Accession: PRJNA1194297): https://www.ncbi.nlm.nih.gov/bioproject/1194297. Further inquiries can be directed to the corresponding authors.
