# Supplementary material for: NECTIN-4-redirected T cell Antigen Coupler T cells bearing CD28 show superior antitumor responses against solid tumors
Source: Front Immunol. 2024 Dec 13;15:1456443. doi: 10.3389/fimmu.2024.1456443 (PMC11681620; doi:10.3389/fimmu.2024.1456443)
Supplement: Supplementary file 1 [file DataSheet1.docx]

**Supplementary Data**


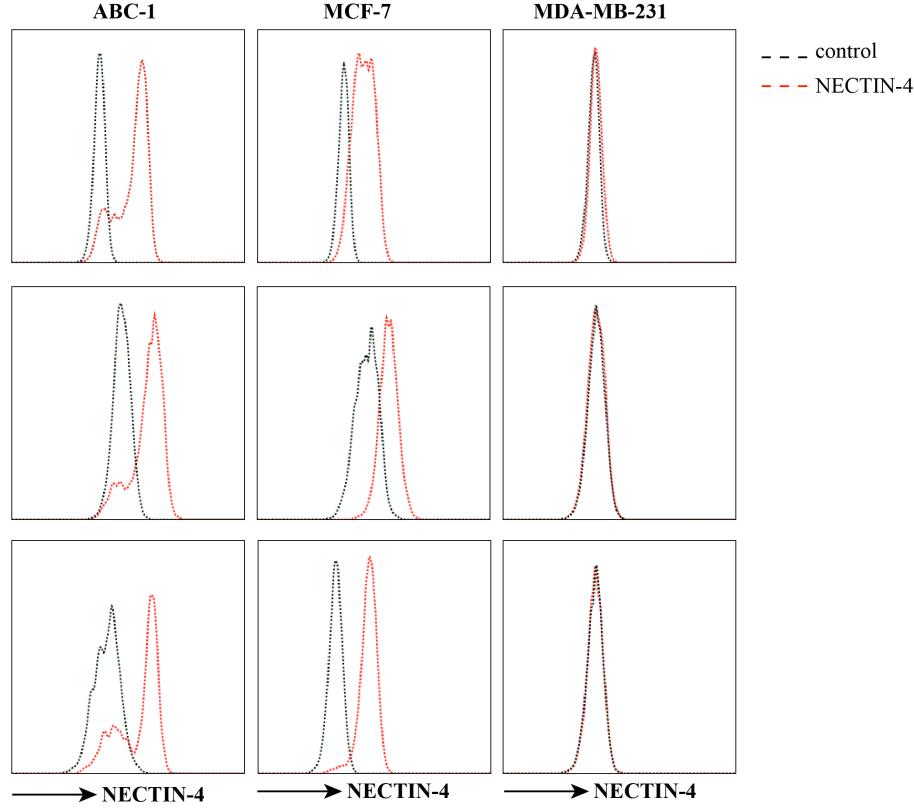


**Supplementary Figure 1.** Expression of NECTIN-4 on ABC-1, MCF-7 and MDA-MB-231 cells was detected by flow cytometry.


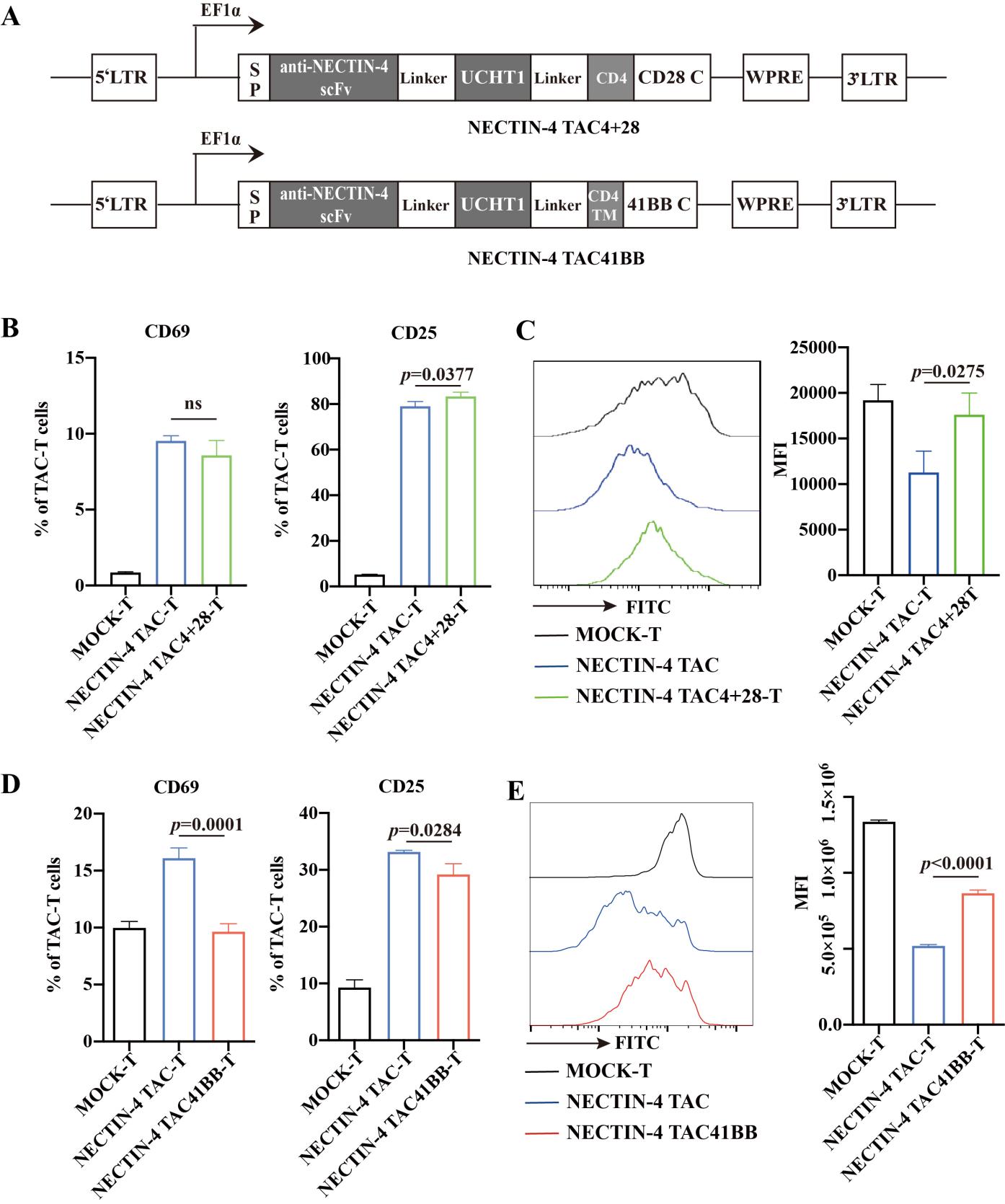


**Supplementary Figure 2.**

**Activation and proliferation of NECTIN-4 TAC4+28-T and NECTIN-4 TAC41BB-T cells.** (A)Schematic illustration of NECTIN-4 TAC4+28 and NECTIN-4 TAC41BB constructs. (B) CD69 and CD25 expression of mock-T, NECTIN-4 TAC-T and NECTIN-4 TAC4+28-T cells upon stimulation with NECTIN-4-beads. (C) Mock-T, NECTIN-4 TAC-T and NECTIN-4 TAC4+28-T cells were labeled with CFSE, and their proliferation were assessed by flow cytometry upon stimulation with NECTIN-4-beads, the left side shows the MFI of CFSE on the surface of T cells, and the right side shows the statistical graph.(D)CD69 and CD25 expression of mock-T, NECTIN-4 TAC-T and NECTIN-4 TAC41BB-T cells upon stimulation with NECTIN-4-beads. (E) Mock-T, NECTIN-4 TAC-T and NECTIN-4 TAC41BB-T cells were labeled with CFSE, and their proliferation were assessed by flow cytometry upon stimulation with NECTIN-4-beads, the left side shows the MFI of CFSE on the surface of T cells, and the right side shows the statistical graph..


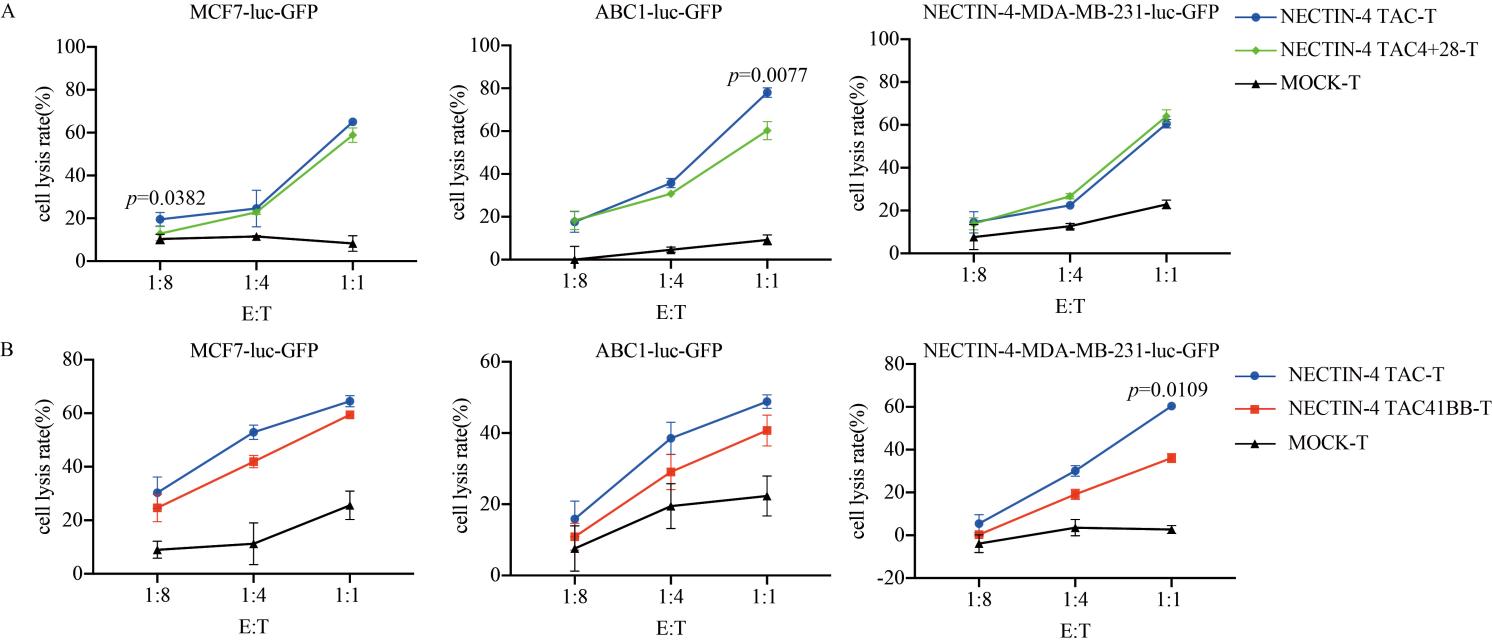


**Supplementary Figure 3.**

**Specific target cell lysis by NECTIN-4-redirected T cells.** (A) Cytotoxicity of NECTIN-4 TAC-T and NECTIN-4 TAC4+28-T cells were assessed by co-incubation with luciferase-expressing MCF7-luc-GFP, ABC1-luc-GFP and NECTIN-4-MDA-MB-231-luc-GFP cells at the indicated E/T ratio. (B) Cytotoxicity of NECTIN-4 TAC-T and NECTIN-4 TAC41BB-T cells were assessed by co-incubation with luciferase-expressing MCF7-luc-GFP, ABC1-luc-GFP and NECTIN-4-MDA-MB-231-luc-GFP cells at the indicated E/T ratio.


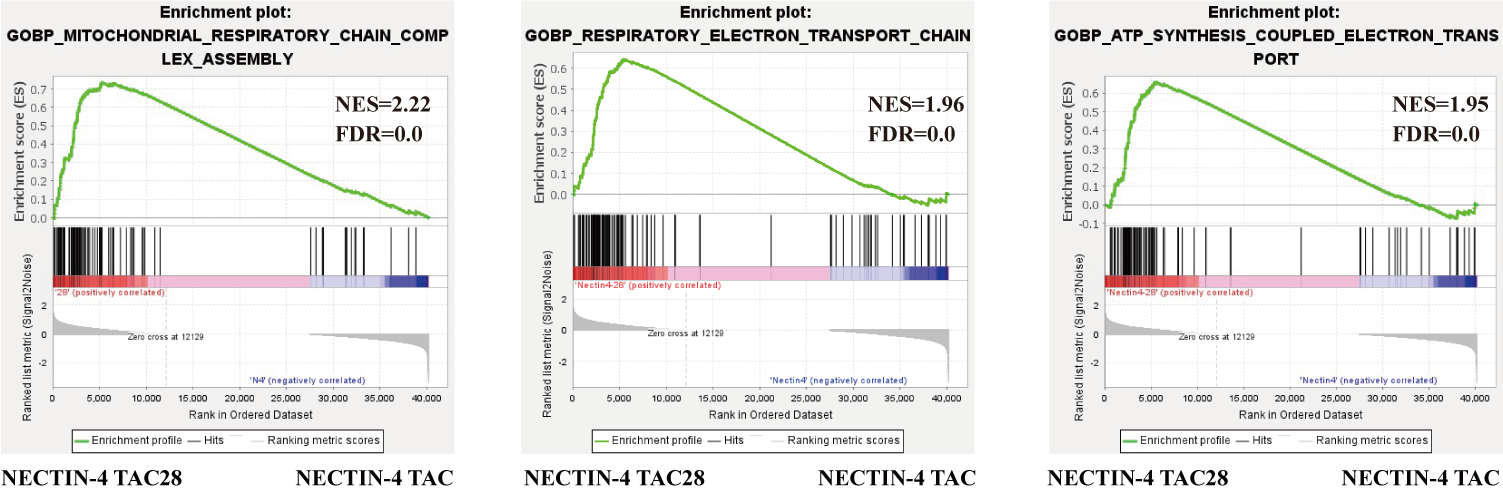


**Supplementary Figure 4.** Representative GSEA results from running the unfiltered NECTIN-4 TAC-T versus NECTIN-4 TAC28-T cell rank list against the MSigDB C5 gene ontology sets.


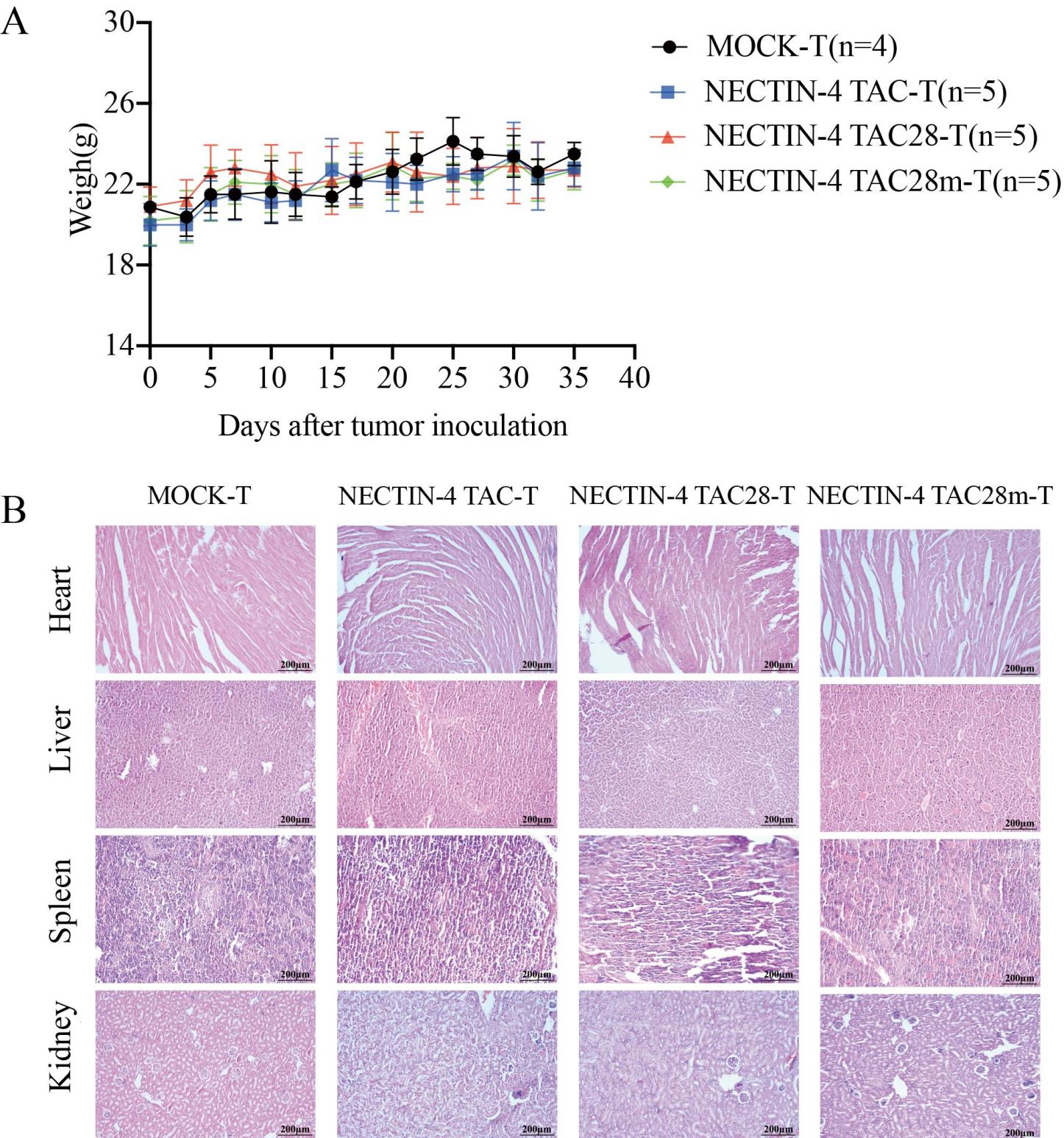


**Supplementary Figure 5.**

**There were no obvious pathological changes in the organs and no weight loss.** (A) Body weight of mice since the tumor inoculation. There were 4 mice in the MOCK-T control group, and 5 mice in each of the other groups. (B) HE staining of important organs in mice.There were 4 mice in the mock-T control group, and 5 mice in each of the other groups.

**
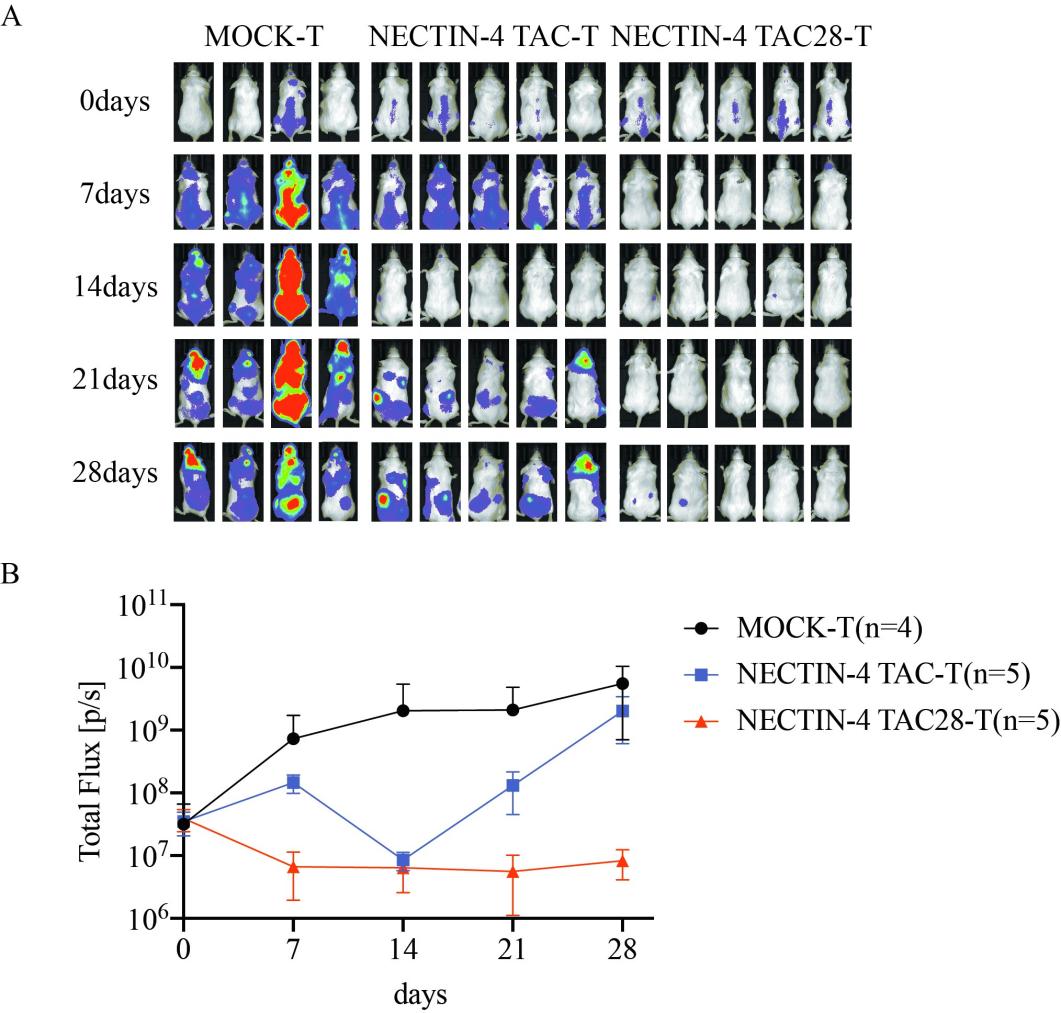
**

**Supplementary Figure 6.**

**NECTIN-4 TAC28-T cells can effectively inhibit the proliferation of NECTIN-4-MDA-MB-231 cells in mice.** 1 million NECTIN-4-MDA-MB-231-luc-GFP cells were injected into mice through the tail vein 7 days in advance.On day 0, mice were treated with MOCK-T cells (n=4), NECTIN-4 TAC-T cells (n=5), and NECTIN-4 TAC28-T cells (n=5), and tumor growth was measured by bioluminescence imaging system at different times such as day 7 and day 14 after treatment.(A)Tumor xenografts were monitored via bioluminescence imaging. (B)Statistical results of fluorescence values in mice at different time points after treatment.


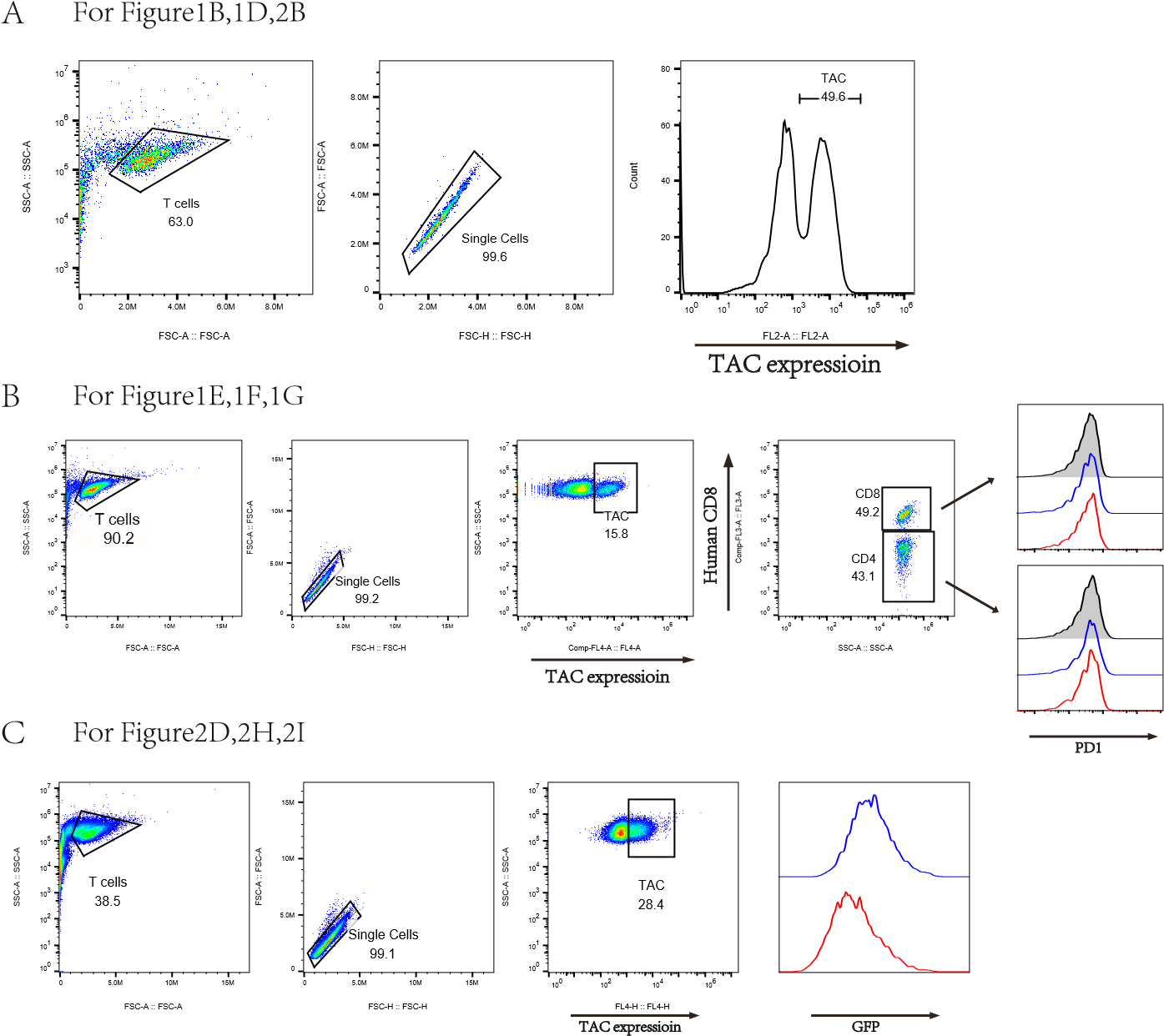


**Supplementary Figure 7.**

**Gating strategy for flow cytometry analysis.** (A) Gating strategy for Figure 1B, 1D, 2B. Lived T cells were gated, then single cells were selected to identify the TAC expression, the gating of TAC positive population was according to Control T cells. (B) Gating strategy for Figure 1E, 1F, 2G. Lived T cells were gated, then single cells were selected to identify the TAC expression, then human CD8 positive cells in TAC-T cells were selected, then PD1 expression were selected. (C) Gating strategy for Figure 2D, 2H,2I. Lived T cells were gated, then single cells were selected to identify the TAC expression, then GFP expression were selected.
